# Production and Radioprotective Effects of Pyrroloquinoline Quinone

**DOI:** 10.3390/ijms12128913

**Published:** 2011-12-05

**Authors:** Xiang-Hua Xiong, Yan Zhao, Xin Ge, Shou-Jun Yuan, Jian-Hua Wang, Jing-Juan Zhi, Yan-Xin Yang, Bao-Hua Du, Wan-Jun Guo, Shan-Shan Wang, De-Xuan Yang, Wei-Cai Zhang

**Affiliations:** 1 Laboratory of Microorganism Engineering, Beijing Institute of Biotechnology, Beijing 100071, China; E-Mails: xiongxianghua@hotmail.com (X.-H.X.); gexin88888@sina.com (X.G.); wang_jianhuaa@126.com (J.-H.W.); 2 Central Laboratory, the First Affiliated Hospital of Xiamen University, Xiamen 361003, China; E-Mail: zhaoyan1976@126.com; 3 Department of Pharmacology, Beijing Institute of Radiation Medicine, Beijing 100850, China; E-Mails: yuan_sj@btmail.net (S.-J.Y.); ammsmelab@126.com (W.-J.G); amms60@126.com (S.-S.W.); amms21_82@sina.com (D.-X.Y.); 4 School of Life Science and Biochemical Pharmaceutical, Shenyang Pharmaceutical University, Shenyang 110016, China; E-Mails: zhijingjuan@126.com (J.-J.Z.); yangyanxin427@163.com (Y.-X.Y.); baohua531@126.com (B.-H.D.)

**Keywords:** methylotroph, pyrroloquinoline quinine, radioprotective, hematopoiesis, survival

## Abstract

Pyrroloquinoline quinone (PQQ) was produced by fermentation of the *Methylovorus* sp. MP688 strain and purified by ion-exchange chromatography, crystallization and recrystallization. The yield of PQQ reached approximately 125 mg/L and highly pure PQQ was obtained. To determine the optimum dose of PQQ for radioprotection, three doses (2 mg/kg, 4 mg/kg, 8 mg/kg) of PQQ were orally administrated to the experimental animals subjected to a lethal dose of 8.0 Gy in survival test. Survival of mice in the irradiation + PQQ (4 mg/kg) group was found to be significantly higher in comparison with the irradiation and irradiation + nilestriol (10 mg/kg) groups. The numbers of hematocytes and bone marrow cells were measured for 21 days after sublethal 4 Gy gamma-ray irradiation with per os of 4 mg/kg of PQQ. The recovery of white blood cells, reticulocytes and bone marrow cells in the irradiation + PQQ group was faster than that in the irradiation group. Furthermore, the recovery of bone marrow cell in the irradiation + PQQ group was superior to that in irradiation + nilestriol group. Our results clearly indicate favourable effects on survival under higher lethal radiation doses and the ability of pyrroloquinoline quinine to enhance haemopoietic recovery after sublethal radiation exposure.

## 1. Introduction

Pyrroloquinoline quinone (PQQ), also known as methoxatin, was identified as a cofactor of bacterial dehydorgenases, including methanol dehydrogenase, glucose dehydrogenase, alcohol dehydrogenase, aldehyde dehydrogenase, *etc.* [1–4]. PQQ has multiple physiological functions [5], such as participating in the electron transport [6], enhancing the adaptability of microbes against extreme environments [7], improving the growth of plants [8], and stimulating the production of nerve growth factor [9], which presents wide application prospect in food industry, pharmaceuticals and agriculture. At present, the organic chemical synthesis of PQQ requires many steps taking a very long time and requires difficult steps for the removal of isomers and various other byproducts [10], whereas bacterial production of PQQ is considered to be economical and environmentally friendly. PQQ has been found to be present in various gram-negative bacteria [11]. Methylotrophs were most outstanding for the production of PQQ. *Methylovorus* sp. strain MP688 (CGMCC No.4096) was collected from soil by our lab as a PQQ producer [12]. The complete genome sequence of MP688 has been sequenced and genes involved in PQQ biosynthesis have been isolated and identified [13].

Recently, it is reported that PQQ could scavenge reactive oxygen species (ROS) [14] and protect AGS cells from oxidative stress-induced damage [15]. PQQ can also effectively improve the activities of free radicals scavenging enzymes and decrease the levels of free radicals [16]. Furthermore, PQQ could prevent oxidative stress-induced neuronal death [17]. These results suggest that PQQ acts as radioprotectant by reducing oxidative damage. In this study, we produced PQQ by fermentation of the *methylovorus* sp. MP688 strain and investigated the possible acute radioprotective effect of PQQ *in vivo* on survival and haematopoietic system in total body irradiated mice. The radioprotective effect of PQQ was also compared with that of nilestriol, which is a representative clinically used estrogenic radioprotector.

## 2. Results and Discussion

### 2.1. PQQ Fermentation, Purification and Detection

The *Methylovorus* sp. MP688 batch fermentation was performed in a 3-L fermentor using MM medium. A time-course study of the PQQ concentration in the supernatant revealed a gradual accumulation of PQQ. The yield of PQQ reached for 125 mg/L after 6 days of fermentation. Ameyama *et al*. showed that some methylotrophs were most favorable for study of PQQ production and that more than 10 μg/mL PQQ was produced in broth after 2 days of incubation [18], and van Kleef and Duine reported that a large number of bacteria, including methylotrophs, extracellularly produced PQQ in microgram amounts per mL of broth [19]. For *Hyphomicrobium* sp. strain TK 0441 at suitable medium, the production of PQQ reached approximately 1 mg/mL at 10 days of cultivation [20].

The best conditions for extractions of PQQ from crude medium supertenant were investigated with various methods. PQQ was efficiently extracted by anion-exchange chromatography, rotary evaporation, crystallization and recrystallization.

### 2.2. Survival of Mice After Irradiation

**Figure 1:**
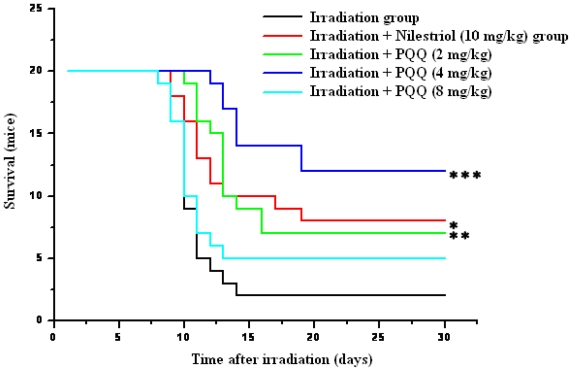
Kaplan–Meier estimate of 30-day survival of 8 Gy gamma-ray irradiated mice in the irradiation, irradiation + nilestriol (10 mg/kg), or irradiation + PQQ (2 mg/kg, 4 mg/kg, 8 mg/kg) group. The respective groups comprised 20 mice. Statistical significance as compared to the irradiation group: * *p* < 0.05, ** *p* < 0.01, *** *p* < 0.001.

For confirmation of the optimum dose of PQQ for radioprotection, three doses (2 mg/kg, 4 mg/kg, 8 mg/kg) of PQQ were orally administrated to the experimental animals subjected to a lethal dose of 8.0 Gy irradiation in survival test. As shown in Figure 1, survival of the mice treated with irradiation + nilestriol (10 mg/kg) or irradiation + PQQ (2 mg/kg, 4 mg/kg) was found to be significantly higher in comparison with that of the mice treated with irradiation. The most significant differences of survival between the irradiation + PQQ (4 mg/kg) group and the irradiation group animals were also observed. Furthermore, survival of the drug group (4 mg/kg PQQ) is also higher than that of positive control group (10 mg/kg nilestriol). Therefore, 4 mg/kg dosage was selected to compare the radioprotective effects of PQQ on hematopoietic function in this study.

If animals were divided by sex into two subgroups, survival of both male and female mice treated irradiation + PQQ (2 mg/kg, 4 mg/kg) was found to be still significantly higher in comparison with that of the mice treated with irradiation. It is the same to the mean surviving time of animals and the mean surviving time of dead animals, but the numerical value of male mice is lower than that of female mice (data not shown). It is accorded with the hypothesis that female may be more resistive against radiation than male because female could synthesize estrogen.

### 2.3. Effect of PQQ on White Blood Cells

Counting of blood cells in the blood of mice irradiated with a sublethal dose of 4.0 Gy revealed effects of the administration of nilestriol or PQQ on white blood cell. As shown in Table 1, mice exhibited a sharp decrease in peripheral white blood cell counts on days 3 after irradiation, and then recovered gradually up to days 21. In comparison with the irradiation group, the number of white blood cell were found to be significantly higher in the irradiation + nilestriol (10 mg/kg) group or irradiation + PQQ (4 mg/kg) group on days 12, 15, 18 and 21, while there was no significant difference in the numbers of white blood cell between irradiation + PQQ group and irradiation + nilestriol group. Like white blood cell, the blood erythrocytes, platelets and hemoglobin drop sharply after irradiation, and then recover gradually until day 21, but no significant differences were found by any comparison among three groups after irradiation (data not shown).

### 2.4. Effect of PQQ on Reticulocytes

Table 2 shows the effect of PQQ on reticulocytes. The percent change of reticulocyte was sharply increased on day 3 after irradiation then recovered gradually till day 21. The level of reticulocyte percentage was found to go back to normal on day 21 in the irradiation + nilestriol and irradiation + PQQ group. In comparison with the irradiation group, the percent change of reticulocyte was found to be significantly lower on days 9, 15, 21 in the irradiation + nilestriol and irradiation + PQQ group.

### 2.5. Effect of PQQ on Bone Marrow Cells

**Figure 2:**
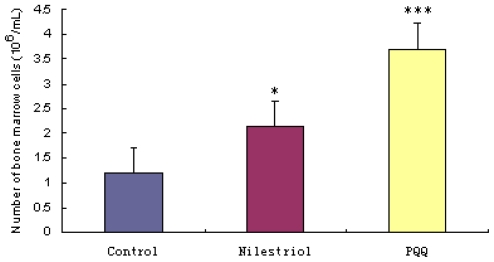
Femoral bone marrow cells on day 21 after irradiation with 4.0 Gy in mice of the irradiation, irradiation + nilestriol and irradiation + PQQ groups. Statistical significance as compared to the irradiation group: * *p* < 0.05, *** *p* < 0.001.

Figure 2 shows that the number of bone marrow cells was significantly lower in the irradiation group ((1.200 ± 0.878) × 10^6^ cells/femur) than that in irradiation + nilestriol group ((2.144 ± 1.052) × 10^6^ cells/femur, *p* < 0.05) and irradiation + PQQ group ((3.711 ± 1.691) × 10^6^ cells/femur, *p* < 0.001) on day 21. There was also significant difference on the number of bone marrow cells in irradiation + PQQ group compared with that of the irradiation + nilestriol group (*p* < 0.05).

Acute effects of high-dose ionizing radiation include hematopoietic cell loss, immune suppression, mucosal damage (gastrointestinal and oral). Its main clinical symptoms depend on the magnitude of absorbed dose [21]. Generally, the death of the irradiated animals was largely attributed to hematopoietic syndrome with irradiation doses of 5–10 Gy, which is characterized by an impaired bone marrow hematopoietic function [22]. Our results show that the administration of PQQ reduced the mice’s radiation-induced mortality, and it apparently did so by protecting hematopoietic system from the effects of irradiation. In general, hematopoietic system (mainly bone marrow tissue and white blood cell) are highly radiosensitive, while erythrocytes, platelets and hemoglobin were less affected by radiation. As can be seen from the results, white blood cell as well as bone marrow cell was significantly protected with 4 mg/kg dose of PQQ against irradiation.

In addition to the results discussed above, the effect of PQQ was compared with that of nilestriol in the present study. Nilestriol, a prolonged-action derivative of estriol, is thought to be a drug against radiation injury [23,24]. Its pharmacological role is to increase leucocyte counts and improve hematopoietic function after irradiation. As a representative of estrogenic radioprotector, nilestriol has been approved by SFDA (State Food and Drug Administration, China) for many years and used widely for antiradiation in clinical therapy. In many pharmacodynamic researches, it was taken as positive control drug [25,26]. In the present study, both nilestriol and PQQ significantly enhanced the recovery of leucocytes, reticulocytes and bone marrow cells in whole body irradiated mice. Moreover, survival of the irradiation + PQQ (4 mg/kg) group is higher than that of the irradiation + PQQ group (*p* < 0.05), and the numbers of bone marrow cells in the irradiation + PQQ group were significantly higher than that in the irradiation + nilestriol group (*p* < 0.05).

The effects of PQQ on whole-body irradiated animals are not fully understood, but one possible mechanism involves its antioxidant properties. It is well known that ionizing radiation produce ROS, which are implicated in the process of DNA damage, cell killing, mutagenesis and carcinogenesis. Therefore, it is reasonable to presume that agents capable of scavenging free radicals would play a significant role in rescuing these processes. The radioprotection of normal cells by a number of synthetic and natural compounds has been reported to be mediated through free radical scavenging activity [27]. PQQ exhibited scavenging activity toward intracellular ROS [25]. The structural analysis of PQQ with other anti-oxidants such as indole and pyrrole derivatives, which act as ROS scavenger, showed that PQQ exhibits comparatively higher reactive electron density, making it a relatively strong antioxidant [28]. Using nanosecond pulse radiolysis technique, PQQ was shown to react with radiolytically produced reactive oxygen species, such as superoxide radicals and hydroxyl radicals *in vitro* [29]. In solution, PQQ protected plasmid DNA from nicking and proteins from oxidative damage caused by gamma-rays [29]. Research results also indicated that PQQ has a regulatory role in the resistance of *Deinococcus radiodurans* to oxidative stress from radiation and DNA-damaging agents [14]. Transgenic *E. coli* cells producing PQQ showed decreased levels of protein oxidation during oxidative stress [30]. These results suggest that PQQ acts as ROS scavenger by directly neutralizing the reactive species and protects the bacterial cells from oxidative stress. PQQ has been also indicated to protect oxidative stress-induced neuronal death [17] and prevent cognitive deficit caused by oxidative stress in mice [31] and scavenge free radicals in skin and serum of SD rats after linear accelerator radiation acute skin injury [16]. However, the molecular and cellular mechanism for the radioprotective effects of PQQ remains to be determined.

## 3. Experimental Section

### 3.1. Bacteria and Chemicals

The *Methylovorus* sp. MP688 strain (CGMCC No. 4096) was isolated from soil by our lab as a PQQ producer. The MM medium contained the following components: MgSO_4_·7H_2_O 0.2 g/L, (NH_4_)_2_SO_4_ 3.0 g/L, KH_2_PO_4_ 1.4 g/L, Na_2_HPO_4_·12H_2_O 3.0 g/L, C_6_H_5_O_7_Fe·5H_2_O 30 mg/L, MnCl_2_·4H_2_O 5.0 mg/L, CaCl_2_·2H_2_O 30 mg/L, ZnSO_4_·7H_2_O 5.0 mg/L, CuSO_4_·5H_2_O 0.5 mg/L, CH_3_OH 1% (v/v). The DEAE Sepharose F.F. column used was obtained from GE Inc. The C18 reverse-phase column was purchased from Dalian institute of chemical physics, Chinese academy of sciences. PQQ standard was purchased from Sigma Chemical Corporation and stock solutions were prepared in water. Nilestriol was bought from Shanghai New Hualian pharmaceutical Co., Ltd. (State medical permit No. H31021647). All other chemicals used were of analytical reagent grade quality or better and were obtained from the usual commercial sources.

### 3.2. Batch Cultivation of MP688 in a 3-L Bioreactor

Batch fermentation of the *Methylovorus* sp. MP688 was performed in a 3 L fermentor (BioStat B5, B. Braun Biotech International, Melsungen, Germany). Seed culture for bioreactor was started from fresh colony that grew on MM plate and was inoculated directly into 500 mL flasks (100 mL working volume) containing MM medium. After 72 h of growth (30 °C, 200 rpm), seed culture was used to inoculate into the bioreactor. 100 mL inoculum (4% v/v) was used for the inoculation of 3 L bioreactor containing 2.5 L medium. Dissolved oxygen was maintained at over 85% air saturation and culture temperature was controlled at 30 °C with pH set at 5.8. The fermentation process was terminated when PQQ stopped accumulating in the culture supernatant.

### 3.3. PQQ Purification

The medium was applied to a DEAE Sepharose F.F. anion-exchange column at a flow rate of 1 mL/min after centrifugation for 30 min at 2500 g, 4 °C. The column was equilibrated with 20 mM sodium citrate buffer, pH 5.5. PQQ was eluted from the column using 20 mM sodium citrate buffer, 1.0 M NaCl, pH 5.5. The fractions containing PQQ were pooled to one fourth volume for further purification by rotary evaporation. The pooled solution was crystallized at 4 °C overnight and the collected crystalline was further recrystallized. The final crystalline was dissolved with a 0.1 M potassium phosphate buffer (pH 7.0) and saved for HPLC analysis.

### 3.4. PQQ Analysis

Samples of PQQ (20 μL) or standard PQQ solution were injected onto the reverse-phase C_18_ column and eluted at a flow rate of 1.0 mL/min with a 70:30 mixture of H_2_O/MeOH (v/v). The high-pressure liquid chromatography analyses were performed with a Waters^TM^ 600 pump and a Waters 600 controller. Eluted PQQ was detected with a Waters 2996 photodiode array detector with detection wavelength of 254 nm. Absorption spectra were measured on a Unico UV2102 spectrophotometer with samples of 2 mm light path.

### 3.5. Animals

KM strain mice with weight of 16–18 g were purchased from the Animal Center, Academy of Military Medical Sciences. These mice were selected and divided into different groups (20 mice per group), namely, irradiation, irradiation + PQQ, irradiation+nilestriol groups, and ten mice were housed in each cage. All animals were kept under controlled lighting conditions (light/dark 12 h/12 h) at a temperature of 25 ± 1 °C and relative humidity of 40%–70%.

### 3.6. Irradiation

All mice were irradiated with single whole body doses using a ^60^Co gamma-ray source (Institute of radiation medicine, Academy of Military Medical Sciences) at a dose rate of 236.05 cGy/min. The mice were placed in ventilated cages and groups of ten mice were irradiated simultaneously. The mice were irradiated with a total dose of 8 Gy gamma-rays in survival test and 4 Gy gamma-rays in haemopoiesis test.

### 3.7. Administration

Nilestriol and PQQ were dissolved in saline and taken orally by mice for all experiments. In survival test, for the selection of the optimum dose of PQQ for radioprotection, three doses (2 mg/kg, 4 mg/kg, 8 mg/kg) of PQQ were administrated to the experimental animals 1 h before gamma irradiation and once daily for 7 consecutive days after gamma irradiation. The control animals received the same volume of saline or nilestriol (10 mg/kg) at 1 h before gamma irradiation and 1 day after gamma irradiation. Twenty mice were used for each treatment group, female and male evenly. In haemopoiesis test, mice were divided into 3 equal groups: PQQ 4 mg/kg, nilestriol 10 mg/kg and control. The administration of drug is the same as that in survival test.

### 3.8. Survival

In experiments evaluating the morality of animals, the survival time was recorded in daily intervals for 30 days after lethal irradiation.

### 3.9. Haematological Methods

In experiments evaluating hematocytes, blood samples were collected from the tail vein on days 0, 3, 6, 9, 12, 15, 18, 21 after sublethal irradiation. At each harvest time, samples were taken from ten mice per group, and the results were averaged over these ten animals. The other ten mice were handled at the next harvest time. The fluctuation of hemograms was automatically counted by a hematocyte counter (Sysmex F820 hematology analyser, Sysmex, Milton, UK) on all days of sampling. Mice were killed by cervical dislocation on days 21, and their femurs were dissected. Bone marrow cells were flushed with PBS and counted using a hematocytometer. The results are expressed as the number of live bone marrow cells (10^6^)/femur.

### 3.10. Statistic Analysis

The data were expressed as mean ± standard error. Statistical analyses of numeration data were performed using analysis of *χ*^2^ text, while Statistical analyses of measurement data were performed using analysis of Student’s *t*-test. Survival data were analyzed by life-table method using the Kaplan-Meier method. The significance level was set at *p* < 0.05.

## 4. Conclusions

In this study, we reported the production and extraction of PQQ. The results showed that the highest yield of PQQ reached 125 mg/L at day 6, and it was efficiently extracted by chromatography, crystallization and recrystallization. Animal test suggested that PQQ exerted significant protection to mice against irradiation injury and this effect was associated with the recovery of hematopoietic function.

## Figures and Tables

**Table 1:** Effect of PQQ (4 mg/kg) on white blood cell in mice exposed to 4 Gy gamma irradiation (%). In comparison with the irradiation group, the number of white blood cell were found to be significantly higher in the irradiation + nilestriol group or irradiation + PQQ group on days 12, 15, 18 and 21, while there was no significant difference in the numbers of white blood cell between the latter two. Statistical significance as compared to the irradiation group:

Group	Time of Irradiation (days)							

	0	3	6	9	12	15	18	21
Irradiation	1.00 ± 0.04	0.11 ± 0.04	0.26 ± 0.12	0.32 ± 0.13	0.42 ± 0.09	0.49 ± 0.12	0.55 ± 0.11	0.76 ± 0.22
Irradiation + nilestriol	1.00 ± 0.02	0.12 ± 0.04	0.19 ± 0.11	0.28 ± 0.14	0.54 ± 0.13 *	0.67 ± 0.22 *	0.70 ± 0.16 *	0.98 ± 0.32 *
Irradiation + PQQ	1.00 ± 0.05	0.13 ± 0.04	0.19 ± 0.05	0.28 ± 0.09	0.54 ± 0.13 *	0.61 ± 0.08 *	0.74 ± 0.16 **	0.91 ± 0.16 *

* *p* < 0.05,

** *p* < 0.01.

**Table 2:** Effects of PQQ (4 mg/kg) on the percent change of reticulocytes (%) in mice exposed to 4 Gy gamma irradiation. In comparison with the irradiation group, the percent change of reticulocyte was found to be significantly lower on days 9, 15, 21 in the irradiation + nilestriol and irradiation + PQQ group. Statistical significance as compared to the irradiation group:

Group	Time of Irradiation (days)				

	0	3	9	15	21
Irradiation	0.32 ± 0.16	4.62 ± 0.89	3.46 ± 0.66	1.63 ± 0.52	0.77 ± 0.43
Irradiation + nilestriol	0.35 ± 0.21	4.55 ± 0.74	1.18 ± 0.61 *	0.94 ± 0.32 *	0.38 ± 0.18 *
Irradiation + PQQ	0.40 ± 0.29	4.07 ± 0.89	0.91 ± 0.33 **	0.90 ± 0.32 *	0.41 ± 0.17 *

* *p* < 0.05,

** *p* < 0.01.
